# Stroke Related Brain–Heart Crosstalk: Pathophysiology, Clinical Implications, and Underlying Mechanisms

**DOI:** 10.1002/advs.202307698

**Published:** 2024-02-02

**Authors:** Xuehui Fan, Jianyang Cao, Mingxia Li, Dechou Zhang, Ibrahim El‐Battrawy, Guiquan Chen, Xiaobo Zhou, Guoqiang Yang, Ibrahim Akin

**Affiliations:** ^1^ Key Laboratory of Medical Electrophysiology Ministry of Education and Medical Electrophysiological Key Laboratory of Sichuan Province Collaborative Innovation Center for Prevention of Cardiovascular Diseases Institute of Cardiovascular Research Southwest Medical University Luzhou 646000 China; ^2^ Cardiology Angiology Haemostaseology and Medical Intensive Care Medical Centre Mannheim Medical Faculty Mannheim Heidelberg University 68167 Heidelberg Germany; ^3^ European Center for AngioScience (ECAS) German Center for Cardiovascular Research (DZHK) Partner Site Heidelberg/Mannheim and Centre for Cardiovascular Acute Medicine Mannheim (ZKAM) Medical Centre Mannheim Heidelberg University 68167 Heidelberg Germany; ^4^ School of Physical Education Southwest Medical University Luzhou Sichuan Province 646000 China; ^5^ Acupuncture and Rehabilitation Department The Affiliated Traditional Chinese Medicine Hospital of Southwest Medical University Luzhou 646000 China; ^6^ Department of Neurology The Affiliated Traditional Chinese Medicine Hospital of Southwest Medical University Luzhou 646000 China; ^7^ Department of Cardiology and Angiology Ruhr University 44780 Bochum Germany; ^8^ Institut für Forschung und Lehre (IFL) Department of Molecular and Experimental Cardiology Ruhr‐University Bochum 44780 Bochum Germany

**Keywords:** arrhythmia, brain–heart crosstalk, cerebrovascular dysfunction, ischemic stroke, pathophysiological mechanisms

## Abstract

The emergence of acute ischemic stroke (AIS) induced cardiovascular dysfunctions as a bidirectional interaction has gained paramount importance in understanding the intricate relationship between the brain and heart. Post AIS, the ensuing cardiovascular dysfunctions encompass a spectrum of complications, including heart attack, congestive heart failure, systolic or diastolic dysfunction, arrhythmias, electrocardiographic anomalies, hemodynamic instability, cardiac arrest, among others, all of which are correlated with adverse outcomes and mortality. Mounting evidence underscores the intimate crosstalk between the heart and the brain, facilitated by intricate physiological and neurohumoral complex networks. The primary pathophysiological mechanisms contributing to these severe cardiac complications involve the hypothalamic‐pituitary‐adrenal (HPA) axis, sympathetic and parasympathetic hyperactivity, immune and inflammatory responses, and gut dysbiosis, collectively shaping the stroke‐related brain–heart axis. Ongoing research endeavors are concentrated on devising strategies to prevent AIS‐induced cardiovascular dysfunctions. Notably, labetalol, nicardipine, and nitroprusside are recommended for hypertension control, while β‐blockers are employed to avert chronic remodeling and address arrhythmias. However, despite these therapeutic interventions, therapeutic targets remain elusive, necessitating further investigations into this complex challenge. This review aims to delineate the state‐of‐the‐art pathophysiological mechanisms in AIS through preclinical and clinical research, unraveling their intricate interplay within the brain–heart axis, and offering pragmatic suggestions for managing AIS‐induced cardiovascular dysfunctions.

## Graphical abstract

Brain–heart crosstalk assumes significance in the aftermath of ischemic stroke. Delving into the underlying mechanisms of this intricate interplay offers a promising strategy for addressing both the stroke and its associated cardiac complications.

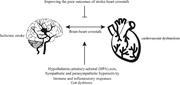

## 1 Background

Acute ischemic stroke (AIS) is a devastating disease for individuals despite remarkable advantages in its diagnosis and treatment and the repercussions extend beyond the affected individuals to encompass their families and the broader social health system. While neurological injury remains a primary cause of stroke‐induced mortality, cardiovascular complications constitute a substantial additional contributor to post‐stroke deaths.^[^ ^1^ ^]^ Common causes of death in AIS patients include cardiovascular diseases, heart failure, pneumonia, and respiratory illnesses.^[^ ^2^, ^3^, ^4^ ^]^ Major adverse cardiovascular complications are markedly elevated after a stroke, correlating with a significantly worsened 5‐year prognosis.^[^ ^5^ ^]^ These cardiac dysfunctions not only exacerbate existing cerebral injury but also contribute to the onset of new brain dysfunctions. A growing body of evidence supports a causal relationship between various brain injuries, including AIS and intracerebral hemorrhage, and heart dysfunction. Hur and Choi reported that approximately 20%−40% of all stroke cases can be attributed to cardiovascular origin.^[^ ^6^ ^]^ Cardiovascular diseases (CVD), including atrial fibrillation (AF), hypertension, coronary heart disease, and cardiac failure, are recognized as primary predisposing risk conditions for AIS.^[^ ^7^, ^8^ ^]^ The occurrence of AIS is fivefold in patients with AF, fourfold for those with cardiac failure, trebled in the presence of hypertension and doubled for those with coronary heart disease, as demonstrated by a 34‐year follow‐up in a Framingham Study.^[^ ^7^ ^]^ Notably, Fuhrer et al. found that while AIS directly impairs cerebral perfusion, cerebral blood flow is intricately linked to cardiac function.^[^ ^9^ ^]^ The emerging field of neurocardiology underscores a two‐way interaction or crosstalk between the brain and the heart.^[^ ^10^, ^11^, ^12^ ^]^ This review accentuates the implicated pathophysiological mechanisms and their clinical consequences following AIS in both preclinical and clinical research. Additionally, it offers detailed proposals for treating CVD after AIS.

## 2 Cardiovascular Dysfunction Following AIS

Cardiovascular complications are prevalent, occurring in over 50% of cases with asymptomatic coronary stenosis and 3% with myocardial infarction within one year in patients without a cardiac history after AIS.^[^ ^13^ ^]^ Prosser et al. highlighted that the more serious cardiac events following a stroke are acute, enabling the identification of patients at their highest risk phase. This understanding facilitates the implementation of more aggressive strategies to improve survival.^[^ ^11^, ^14^ ^]^ The risk of developing cardiac complications is commonly observed following AIS.^[^ ^15^ ^]^ Similarly, the impaired cardiac function, objectively determined left ventricular ejection fraction (LVEF), exhibits comparable predictive ability for AF and 90‐day disability in stroke patients, as opposed to commonly defined heart failure.^[^ ^16^ ^]^ 76% of patients with severe autonomic dysfunction, identified via Ewing battery after AIS, show a poorer functional outcome.^[^ ^17^ ^]^ Following AIS, 23.6% develop AF, 10.4% develop systolic dysfunction, and 24.5% develop diastolic dysfunction.^[^ ^18^ ^]^ The study from Adeoye et al. found that a third of 1020 adult patients with acute stroke had systolic dysfunction, highly associated with 1‐month mortality.^[^ ^19^ ^]^ Electrocardiogram (ECG) abnormalities within the initial 24 hours are frequently observed in 85% of acute stroke patients.^[^ ^20^, ^21^ ^]^ However, Chen et al. found that individuals aged 65 to 80 years with documented paroxysmal atrial fibrillation (AF) on ECG did not have a stroke.^[^ ^22^ ^]^ Other studies have demonstrated the prevalence of abnormal ECG findings among elderly individuals.^[^ ^23^, ^24^, ^25^ ^]^ The clinical utility of ECG in ischemic stroke patients is indicated to be limited, necessitating the consideration of other symptoms for a comprehensive evaluation. To sum up, despite these observations, Sposato et al., based on a large population‐based study, suggested that the explanation for this association—whether it is due to stroke‐associated cardiac injury, preexisting subclinical cardiovascular comorbid conditions, or both—remains unknown.^[^ ^26^, ^27^ ^]^

Upon searching for the clinical trials on cardiovascular dysfunctions following ischemic stroke, the results are presented in **Table** **1**. The table indicates that clinical trials on the cardiovascular dysfunctions of ischemic stroke are currently in a rudimentary stage.

**Table 1:** Recent studies, encompassing observational and randomized studies, have extensively explored the occurrence and frequency of cardiovascular dysfunctions following ischemic stroke.

Author	Year	Journal	Incidence, prevalence, therapeutic strategy
			Autonomic dysfunction
Diserens et al.[^28^ ^]^	2006	Eur J Neurol	Acute autonomic dysfunction was present in 71% of acute stroke patients.
Xiong et al.[^29^ ^]^	2012	Clin Neurol Neurosurg	The prevalence of severe autonomic dysfunction in ischemic stroke patients was 76.5%.
Nayani et al.[^30^ ^]^	2016	Clin Neurol Neurosurg	21.8% of patients developed autonomic dysfunction.
Xiong et al.[^17^ ^]^	2018	Stroke	Minor (24%) and significant autonomic dysfunction (76.0%) were identified in patients with ischemic stroke.
Chang et al.[^31^ ^]^	2020	Biol Res Nurs.	Heart rate variability biofeedback is a promising intervention for improving autonomic function, cognitive impairment, and psychological distress in AIS patients.
Wei et al.[^32^ ^]^	2020	Complement Ther Med.	30‐day remote ischemic postconditioning could further autonomic function by enhancing the vagus nerve activity and autonomic nerve activity in AIS patients
			Arrhythmias
Dogan et al.[^33^ ^]^	2004	Anadolu Kardiyol Derg	ECG abnormalities were found in 65% of patients with ischemic stroke. AF (34%) was more frequent in stroke patients.
Christensen et al.[^20^ ^]^	2005	J Neurol Sci	ECG abnormalities: 60% in ischemic stroke.
Ritter et al.[^34^ ^]^	2011	BMC Neurol	Tachycardia was found in 5% of patients with ischemic stroke, and bradycardia in 5%.
Kallmunzer et al.[^35^ ^]^	2012	Stroke	Significant cardiac arrhythmias occurred in 25.1% of patients with ischemic stroke.
Gonzalez‐Toledo et al.[^36^ ^]^	2013	J Stroke Cerebrovasc Dis	New AF was seen in 8.36%, known AF in 23.27%, and normal sinus rhythm in 68.36%.
Purushothaman et al.[^37^ ^]^	2014	J Nat Sci Biol Med	Electrocardiographic changes were noted in 78% of stroke patients.
.Bobinger et al.[^38^ ^]^	2015	Clin Res Cardiol	Early repolarization pattern was found in 8.04% of stroke patients.
Yayehd et al.[^39^ ^]^	2015	Arch Cardiovasc Dis	Paroxysmal AF lasting >30 s, paroxysmal AF lasting < 30 s, numerous premature atrial complexes, and non‐sustained ventricular tachycardia were confirmed during monitoring.
Hromadka et al.[^40^ ^]^	2016	J Stroke Cerebrovasc Dis	Incidence of prolonged QTc was 65.2% at baseline and 26.1% after 48 h.
Adeoye et al.[^21^ ^]^	2017	Glob Heart	ECG abnormalities were noted in 85.4% of patients with ischemic stroke.
Wachter et al.[^41^ ^]^	2017	Lancet Neurol	AF was found after 6 months in 14% of 200 patients of the enhanced and prolonged monitoring group compared with 5% in the standard care group.
Tanislav et al.[^42^ ^]^	2019	Eur J Neurol	AF at 5‐year follow‐up occurred in 10.4% of stroke patients.
			Myocardial infarction and coronary artery disease (CAD)
Leys et al.[^43^ ^]^	2006	Cerebrovasc Dis	Among 753 patients with ischemic stroke, 16% had a previous coronary event, Atherothrombosis was found in 47.5% of patients (33.7% coronary artery, 16.6% aortic atheroma, and 22.7% peripheral artery disease).
Nighoghosian et al.[^44^ ^]^	2006	Eur Neurol	Dobutamine stress echocardiography was positive in 15% of 64 patients with ischemic stroke. The main predictive factor for a positive dobutamine stress test was aortic arch atheroma (*p* = 0.003).
Seo et al.[^45^ ^]^	2008	Eur Neurol	Coronary artery stenosis was detected in 25.4% of 89 patients with ischemic stroke. Intracranial arterial stenosis was not associated with coronary stenosis.
Lee et al.[^46^ ^]^	2008	Neurocrit Care	Of 1357 ischemic stroke patients, 0.9% developed myocardial infarction. Five patients died of cardiac complications.
Hoshino et al.[^47^ ^]^	2008	Intern Med	Of 100 patients with ischemic stroke, 36 had CAD.
Liao et al.[^48^ ^]^	2009	Eur J Neurol	Among 9180 ischemic stroke patients, 2.3% had myocardial infarction.
Calvet et al.[^49^ ^]^	2010	Circulation	The prevalence of asymptomatic CAD >50% was 18% (95%CI, 14–23).
Arauz et al.[^50^ ^]^	2010	Clin Neurol Neurosurg	Among 125 patients with ischemic stroke, silent CAD was identified in 32%, of whom 30% had single lacunar infarction, 33% had multiple lacunar infarction, and 33% had a large‐vessel stroke. The stroke recurrence rate was 8%.
Yoon et al.^[^ ^51^ ^]^	2011	Int J Cardiovasc Imaging	Of 175 patients with ischemic stroke, 60% had demonstrable atherosclerotic plaques; 21% had occult CAD with >50% stenosis.
Cho et al.^[^ ^52^ ^]^	2011	Cerebrovasc Dis	Of 469 patients with ischemic stroke (274 with no history of CAD), 22.3% had asymptomatic CAD with more than 50% stenosis; 1.2% had complicated aortic plaques.
Amarenco et al.^[^ ^53^ ^]^	2011	Stroke	Coronary plaques were found in 61.9% of 864043 patients with ischemic stroke at angiography, and coronary stenosis in 25.7%.
Micheli et al.^[^ ^54^ ^]^	2012	J Neurol	Among 814 stroke patients, 2.25% presented with myocardial infarction, 4.85% developed acute heart failure, and 2.08% had both myocardial infarction and heart failure.
Kim et al.^[^ ^55^ ^]^	2012	Eur J Neurol	Among 443 ischemic stroke patients, 36% showed asymptomatic CAD based on CT.
Cha et al.^[^ ^56^ ^]^	2013	Eur J Neurol	70.4% of 1733 ischemic stroke patients had CAD.
Gattringer et al.^[^ ^57^ ^]^	2014	Cerebrovasc Dis	1% of 406603 ischemic stroke patients had myocardial infarction. Anterior circulation and left‐sided stroke were more frequent in patients with myocardial infarction.
Mathias et al.^[^ ^58^ ^]^	2014	J Cardiovasc Dis	Of 426 patients with ischemic stroke, 4.9% had myocardial infarction.
Alqahtani et al.^[^ ^59^ ^]^	2017	Stroke	Among 864043 ischemic stroke cases, 1.6% had acute myocardial infarction (79.5% NSTEMI and 20.5% STEMI).
Bhatia et al.^[^ ^60^ ^]^	2019	J Stroke Cerebrovasc Dis	Of 300 ischemic stroke patients, 247 were asymptomatic, of whom 4.81% had a positive myocardial perfusion scan. The overall prevalence of CAD was 17.67%.
			Cardiac dysfunction and remodeling
Burkot et al.^[^ ^61^ ^]^	2015	J Card Fail	Among 566 patients with stroke, decompensated heart failure was diagnosed in 17%. Of these, 57% had preserved ejection fraction.
Kim et al.^[^ ^62^ ^]^	2016	J Stroke Cerebrovasc Dis	Among 1554 patients with AIS, mild left ventricular dysfunction in 5.6%, and severe left ventricular dysfunction in 3.22%.
Choi et al.^[^ ^63^ ^]^	2017	Neurology	Left ventricular wall motion abnormalities occurred in 9.96% of patients with ischemic stroke.
de Havenon et al.^[^ ^64^ ^]^	2019	Stroke	Both systolic and diastolic blood pressure variability (BPV) were associated with myocardial infarction, or new or worsening heart failure.
Verschoof et al.^[^ ^65^ ^]^	2020	Stroke	2124 AIS patients, 1440 patients had a normal systolic blood pressure (SBP) (range, 130–184 mm Hg). Low SBP was associated with an increased risk of in‐hospital mortality. Heart failure was more common in patients with low SBP.

## 3 Clinical Implications of Stroke‐Related Brain–Heart Crosstalk

Cardiac issues, encompassing AF, mural thrombus, patent foramen ovale, infective endocarditis, and hematological disorders, have established associations with AIS.^[^ ^66^ ^]^ Preclinical and clinical evidence indicates that individuals lacking a known cardiac history face a heightened risk of ischemic heart disease within one year following AIS.^[^ ^13^ ^]^ Notably, decompensated heart failure is diagnosed in up to 17% of patients during hospitalization after AIS, representing a robust independent predictor of unfavorable functional prognosis post‐ischemic stroke.^[^ ^61^, ^67^ ^]^ Reports indicate that 18–70% of AIS patients exhibit some degree of CAD.^[^ ^56^, ^60^ ^]^

The physiological stress response and autonomic dysregulation are likely the main causative factors contributing to stroke‐mediated functional and structural alterations, which include microvascular dysfunction, myocardial necrosis, coronary demand ischemia, and arrhythmogenesis in normal neural cardiac dysregulation.^[^ ^68^, ^69^ ^]^ The high prevalence of shared risk factors, such as traditional vascular risk factors, intracranial artery calcifications, cervicocephalic artery stenosis, and atherosclerosis, may explain the frequent co‐occurrence of cardiovascular and cerebrovascular disease, suggesting a reciprocal relationship between the two.^[^ ^49^, ^70^, ^71^, ^72^ ^]^

The incidence of significant cardiac arrhythmias surges within the first day of care for 501 acute neurovascular patients.^[^ ^35^, ^73^, ^74^ ^]^ Autonomic dysfunction,^[^ ^30^, ^75^ ^]^ myocardial infarction,^[^ ^59^, ^76^ ^]^ cardiac arrhythmias,^[^ ^77^ ^]^ takotsubo syndrome (TTS)^[^ ^15^, ^78^ ^]^ and higher BPV,^[^ ^79^, ^80^, ^81^ ^]^ are prevalent clinical features signifying dysregulation of brain–heart axis. Heart rate variability analysis using continuous ECG signals has shown promise in predicting outcomes in patients with acute stroke.^[^ ^82^, ^83^ ^]^ AF, known to induce blood stagnation in the left atrium (LA) and predispose to thrombosis and cerebral embolism, poses a significant risk for AIS.^[^ ^84^, ^85^, ^86^ ^]^ Best et al. reported a 17‐fold increase in the risk of AIS following valvular AF compared to a fivefold increase after non‐valvular AF.^[^ ^84^ ^]^ The risk rate for AIS does not distinguish between paroxysmal, permanent, or persistent AF.^[^ ^84^ ^]^ Elevated systolic blood pressure (SBP) of 140 mmHg or higher resulted in 4.9 million ischemic heart disease and 1.5 million AIS‐related deaths between 1990 and 2015.^[^ ^87^ ^]^ Additionally, due to its high prevalence in public health, hypertension ultimately leads to an increased risk of AF, a recognized risk factor for stroke.^[^ ^88^, ^89^, ^90^ ^]^ Clinical research by Alkhachroum et al. has highlighted that ischemic myocardial infarction with troponin elevation can occur after AIS and is associated with higher mortality.^[^ ^91^ ^]^

Localization of the stroke area emerges as a fundamental predictor of cardiac consequence. A population‐based study in a multiethnic urban population performed by Rincon et al. highlights that parietal lobe infarction can induce myocardial infarction and long‐term cardiac death, serving as an independent risk factor for fatal cardiac outcomes.^[^ ^92^ ^]^ Incidence of prolonged QTc interval, tachyarrhythmias, and the left bundle block elevate following right insular damage.^[^ ^93^ ^]^ Right insular damage, cardiac autonomic dysfunction, and arrhythmias, when integrated into traditional risk stratification, offer a practical approach to identifying stroke patients at early mortality risk.^[^ ^1^, ^94^, ^95^ ^]^ TTS exhibits a strong association with specific ischemic areas in the right anterior circulation, particularly insular and peri‐insular areas.^[^ ^96^ ^]^ Left focal cerebral ischemia, especially the extent of damage to the left insular cortex, correlates with cardiac dysfunction. Furthermore, except for exhibited cardiac dysfunction in stroke mice, elevated levels of norepinephrine can be observed in the serum and heart.^[^ ^97^ ^]^

It has been reported that cardiac remodeling is a common consequence accompanied by arrhythmogenesis or chronic myocardial changes in the recent understanding of heart disease.^[^ ^98^ ^]^ One preclinical research employing young and aged mice with transient middle cerebral artery occlusion (MCAO) demonstrated that focal AIS and its subsequent increased sympathetic activity are succeeded by chronic cardiac dysfunction and remodeling, as analyzed through hemodynamic measurements and serial transthoracic echocardiography.^[^ ^1^, ^14^, ^99^ ^]^ A clinical trial conducted by Fonseca et al. revealed that patients with undetermined stroke significantly develop structural or functional changes, including chronic atrial fibrosis of the LA and left atrial appendage (LAA), based on a 3T cardiac magnetic resonance imaging (CMRI), suggesting an association between atrial disease and stroke.^[^ ^100^, ^101^ ^]^ TTS, predominating in women following AIS, is associated with left ventricular dysfunction, leading to short‐term poor functional outcomes at the apex (neurological deterioration, high mortality, and functional status at discharge), with changes in laboratory findings and radiological features.^[^ ^15^, ^96^ ^]^ It is mainly reversible and typically self‐limiting due to neurogenic activation and clinical management is centered on supportive treatment.^[^ ^102^ ^]^ After AIS, systolic and diastolic ventricular dysfunctions are considered the main complications.^[^ ^103^ ^]^

**Figure 1:**
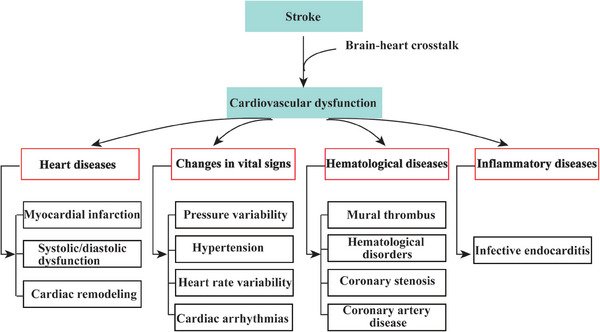
AIS‐related cardiovascular dysfunction. There are different types of cardiovascular dysfunction (heart diseases, vital sign changes, hematological diseases, and inflammatory diseases) following AIS, including myocardial infarction, autonomic dysfunction (higher sympathetic and parasympathetic activity), systolic or diastolic dysfunction, cardiac remodeling, arrhythmogenesis (AF, prolonged QTc interval, the risk of tachyarrhythmias, and the left bundle block, etc.), etc. These cardiovascular diseases always follow the AIS onset and these diseases also induce the AIS happening. Abbreviations: AIS, acute ischemic stroke; AF, atrial fibrillation; ECG, electrocardiogram.

Hence, given the robust accumulation of evidence on the brain–heart interaction above, it is critical to identify cardiovascular events to effectively control the poor outcomes following AIS (**Figure** **1**).

## 4 Pathophysiological Mechanisms Following Stroke‐Heart Interaction

Key mechanisms contributing to the intimate crosstalk between the heart and the brain in stroke‐heart interactions include the hypothalamic‐pituitary‐adrenal (HPA) axis,^[^ ^104^ ^]^ immunity and inflammation,^[^ ^105^ ^]^ atherosclerosis, microbiota‐immune axis and neurohumoral system (autonomic nervous system, the limbic network, and the renin‐angiotensin‐aldosterone system (RAAS)).^[^ ^106^, ^107^, ^108^, ^109^ ^]^ Both unmodifiable such as age, sex, race, and modifiable risk factors like physical inactivity, diet, diabetes, dyslipidemia, hypertension, and smoking are associated with stroke‐heart crosstalk.^[^ ^109^, ^110^ ^]^ Preclinical and clinical studies highlight a higher baseline sympathetic activity under physiological conditions in men. In contrast, while women exhibit a more pronounced parasympathetic tone for maintaining sympathovagal balance. Interestingly, this difference attenuates with increasing age, possibly resulting from changes in sex hormone concentrations affecting the autonomic system at central and peripheral levels.^[^ ^109^ ^]^ These mechanisms and risk factors primarily contribute to AIS pathogenesis. Findings from a cross‐sectional study suggest that HPA axis activity can predict CVD and stroke.^[^ ^111^ ^]^ Additional evidence indicates that inflammation plays a critical role in the brain–heart crosstalk following stroke. The stroke‐evoked systemic inflammatory response may directly damage myocardial cells, where the interaction of inflammation, immunoregulation cardiac, and sympathetic/parasympathetic dysfunction may be the major pathophysiology mechanisms.^[^ ^112^ ^]^ In general, a profound understanding of the potential mechanism in brain–heart crosstalk provides more opportunities for treating related diseases in clinical research.

There is no denying that identifying risk factors for AIS is complicated due to shared similar risk factors with cardiac dysfunction. For instance, hypertension, the most important modifiable risk factor for atherosclerotic disease, plays an essential role in AIS pathophysiology.^[^ ^110^ ^]^ It also contributes to cardiovascular accidents, thereby complicating the interpretation of post‐AIS cardiac dysfunction. Additionally, cerebral vessel embolism originating from an atherosclerotic ulcerated plaque stands out as a leading cause of AIS. Atherosclerotic plaques and minimal vessel disease not only promote lacunar AIS, cerebral hemorrhage, and leukoaraiosis but also contribute to atherosclerotic cardiovascular disease.^[^ ^66^, ^113^, ^114^ ^]^

### 4.1 Hypothalamic‐Pituitary‐Adrenal (HPA) Axis

The HPA axis functions as a central regulator of hormonal balance, orchestrating responses to emotion, stress, physical activity state, and metabolism.^[^ ^111^, ^115^ ^]^ Comprising intricate interactions among three endocrine glands—the hypothalamus, pituitary, and adrenal glands—the HPA axis plays a crucial in the neuroendocrine system. The principal regulatory center of the HPA axis is the hypothalamic paraventricular nucleus, releasing corticotropin‐releasing factors, including corticotrophin‐releasing hormone and vasopressin. These factors stimulate the pituitary gland to release adrenocorticotropic hormone (ACTH), particularly during stressful conditions. ACTH, in turn, stimulates the adrenal gland to release the steroid hormone cortisol. Serum cortisol levels have been associated with stroke severity and extent of insular damage.^[^ ^104^ ^]^ Post‐stroke depression in patients, involving psychological elements such as anxiety, fear, and stress, is both psychological and biochemical, significantly correlating with neurological functional deficits. Many patients experience early‐onset depression linked to the severity of their functional deficits or late‐onset depression.^[^ ^116^, ^117^ ^]^ Ischemic stress can commonly induce TTS in females experiencing insular damage.^[^ ^15^ ^]^ Prolonged cortisol elevation is potentially neurotoxic and has been linked to elevated post‐stroke mortality rates.^[^ ^104^ ^]^ Additionally, the paraventricular nucleus projects directly to the rostral ventrolateral medulla, facilitating the integration of cardiac afferents, baroreceptor activity, and inputs from higher brain regions into sympathetic outflow to the heart. Hypothalamic stimulation triggers sympathetic output, leading to ECG abnormalities, and arrhythmias.^[^ ^118^, ^119^ ^]^ Activation of the paraventricular nucleus in the hypothalamus of rats exposed to ischemic stroke induces glutamate‐mediated arrhythmias via activation of N‐methyl‐D‐aspartic acid receptors.^[^ ^120^ ^]^ Diminishing paraventricular nucleus activity facilitates enhanced recovery of cardiac function following myocardial infarction.^[^ ^121^ ^]^ HPA axis activation results in a notable surge in catecholamines.^[^ ^122^ ^]^ The widely accepted mechanism for brain–heart interaction is the catecholamine surge hypothesis. Experimental animal studies have shown a proportional increase in plasma catecholamine levels following ischemic stroke, correlating directly with the occurrence of myocardial lesions and cardiac damage.^[^ ^123^ ^]^ The autonomic nervous system regulates the release of catecholamines from the adrenal glands. Brain injury can lead to an elevated sympathetic tone, accompanied by increased catecholamine secretion and increased circulating catecholamines from myocardial nerve endings.

### 4.2 Enhanced Sympathetic and Parasympathetic Activity

The forebrain plays a crucial role in patients with AIS, wielding influence over the autonomic nervous system. Noteworthy is its consistent impact on blood pressure and heart rate through the stimulation of the orbital surface of the frontal lobe and the anterior cingulate gyrus. Within the central autonomic network, the insular cortex holds a crucial position.^[^ ^69^ ^]^ Ischemic lesions in the insular cortex elevate the susceptibility of cardiac complications, manifesting in fluctuations in blood pressure, cardiac arrhythmias, and myocytolysis.

In a prospective observational study, 150 adult patients with ischemic stroke within 7 d post‐stroke were stratified based on the magnitude of autonomic dysfunction. The results indicated that autonomic dysfunction, assessed by Ewing battery, can independently predict clinically unfavorable outcomes in patients with AIS.^[^ ^17^ ^]^ Two separate patient cohorts investigated the association between left‐hemisphere brain infarction and heightened mortality.^[^ ^124^ ^]^ The central autonomic nervous system, acting through sympathetic and parasympathetic nerves, plays a pivotal role in conveying central autonomic commands from the brain to the heart and is associated with structures involved in pathophysiology, arousal, and sleep.^[^ ^1^, ^125^, ^126^ ^]^ Sympathetic innervation involves sympathetic preganglionic neurons located in the spinal upper thoracic segment that synapse with sympathetic postganglionic neurons for downward conduction to the heart. Additionally, neurons of the cervical and upper thoracic ganglia contribute to upward conduction to the central nucleus.^[^ ^125^ ^]^ Elevated sympathetic nervous system activity during the acute phase of SAH induces myocardial damage and contributes to the development of cardiac dysfunction. A study by Gilley et al. demonstrated that increased forkhead box class O (FOXO) activity can lead to nerve growth factor (NGF) deprivation‐induced sympathetic neuron death.^[^ ^127^ ^]^ Upstream changes of FOXO proteins (FOXOs) include brain‐derived neurotrophic factor (BDNF) and protein kinase B (PKB), both of which have been implicated in various brain disorders. Furthermore, FOXOs may be influenced by the HPA and serotonin or noradrenaline signaling.^[^ ^128^, ^129^, ^130^ ^]^ FOXO genes play critical roles and represent potential novel therapeutic molecular targets for major cardiac diseases, including myocardial infarction, diabetic cardiomyopathy, and myocardial hypertrophy). Inhibition of FOXO in cardiac myocytes has shown promise in improving the pathogenic phenotype and prolonging survival in a mouse model of laminopathies.^[^ ^131^, ^132^, ^133^, ^134^ ^]^

Parasympathetic connections controlling cardiac function, involve parasympathetic preganglionic neurons located in the medulla oblongata, specifically in the ventrolateral to nucleus ambiguus, dorsal motor nucleus of the vagus nerve, and reticular formation.^[^ ^125^ ^]^ These neurons extensively innervate cardiac parasympathetic postganglionic fibers in the cardiac conduction system, the atrial and ventricular working myocardium. Operating independently of sympathetic activity, these fibers release acetylcholine, a high density of acetylcholine M2 receptors and vasoactive intestinal peptide verified according to acetylcholinesterase staining and choline acetyltransferase immunohistochemistry.^[^ ^125^, ^135^ ^]^ These nuclei, which are connected with the epicardial ganglionated plexus, establish communication with postganglionic fibers.^[^ ^125^ ^]^ By binding to M2 muscarinic receptors in myocardial cells, acetylcholine decreases intracellular cyclic adenosine monophosphate (cAMP) levels. Activating this pathway during parasympathetic activity upregulates atrioventricular conduction time and weakens atrial and ventricular contractility.^[^ ^125^ ^]^

### 4.3 Inflammatory Response

It is increasingly evident that human stroke causes multiorgan systemic disease, resulting in local and systemic effects.^[^ ^136^ ^]^ Blood‐brain barrier (BBB) disruption, associated with poor outcomes, may facilitate increased infiltration of neutrophils and peripheral macrophages into the ischemic brain tissue following AIS. This infiltration plays a crucial role in maintaining the integrity of the neurovascular unit in an ischemic stroke mice model.^[^ ^137^, ^138^, ^139^, ^140^ ^]^ The size of the ischemic area following AIS correlates with the severity of cardiac dysfunction, in which the early local neuroinflammation influences subsequent cardiac dysfunction in ischemic stroke mice and rats. Moreover, disruption of BBB enables the entry of brain‐derived antigens and extracellular vesicles originating from injured brain cells into the bloodstream, where they interact with peripheral immune cells. Consequently, a close relationship exists between cardiac function and BBB integrity, though the effects are likely to be indirect. Additionally, the total body mitochondrial translocator protein, targeted by positron emission tomography imaging, can be utilized to monitor neuroinflammation, providing a foundation for understanding the brain–heart interconnection and developing strategies to preserve cardiac function post‐AIS.^[^ ^141^, ^142^, ^143^ ^]^

The immune‐inflammatory response is strongly associated with the progression of ischemic and hemorrhage stroke, primarily based on an immediate cascade of microglial activation and cell death products.^[^ ^144^, ^145^, ^146^ ^]^ Microglia/macrophages undergo polarization to the M1‐phenotype, traversing the damaged BBB and being attracted into the brain tissue injured site.^[^ ^146^, ^147^ ^]^ Subsequently, an intensified local inflammatory response is triggered, characterized by elevated levels of cytokines, chemokines, astrogliosis, microgliosis, and endothelial cell activation.^[^ ^146^ ^]^ Concurrently, elevated levels of matrix metalloproteinases (MMP) and oxidative stress contribute to endothelial cell damage post‐stroke, further aggravating BBB injury and subsequent formation of vasogenic edema.^[^ ^148^, ^149^ ^]^ Consequently, peripheral immune cells are recruited into the cerebral microcirculation and ischemic brain tissue, subsequently crossing the damaged BBB through activation of the sympathetic nervous system and the HPA axis.^[^ ^136^ ^]^ Following ischemia, the brain releases danger‐associated molecular patterns (DAMP), activating Toll‐like receptors (TLRs) and recruiting peripheral immune cells to clear cell debris and facilitate brain healing.^[^ ^136^ ^]^ The local inflammatory response extends into the systemic circulation, potentially causing secondary cardiac damage. An in vitro experiment demonstrated that rat heart myocytes exposed to supernatant from 90 min of oxygen‐glucose deprivation of primary rat neuronal cells exhibited a significant reduction in cell viability and mitochondrial activity, and in vivo study also found phenotypic expression of necrosis, apoptosis, and autophagy in cardiac myocytes, paralleled by the cell death markers in their brain tissue.^[^ ^150^ ^]^ Recent preclinical and clinical reports have demonstrated neural and cardiac myocyte cell death after AIS, indicating a close pathological link between stroke in the brain and cardiac dysfunction.^[^ ^99^, ^103^, ^151^ ^]^ Chronic secondary injuries, marked by aberrant inflammation and apoptosis, were observed in the ischemic cerebellum with significant Purkinje cell loss, hippocampus with CA1 cell loss, and heart of non‐human primates relative to sham brain and hearts 6 months after transient global ischemia induction.^[^ ^152^, ^153^ ^]^ This underscores the importance of developing therapeutic interventions targeting both the brain and the heart to mitigate cerebral ischemia and its associated comorbidities.

Increasing evidence suggests that following ischemic stroke, microglia, astrocytes, and oligodendrocytes collectively participate in the pathophysiological mechanisms, contributing to the correction of neural tissue functioning.^[^ ^154^, ^155^ ^]^ Under homeostatic circumstances, astrocytes remain dormant and change reactive in response to various CNS pathologies, including stroke, neurodegenerative diseases, and traumatic injury.^[^ ^156^ ^]^ Astrocyte reactivity is a well‐documented physiological response, involving phenotypic and molecular changes, aimed at restoring neurological function and homeostasis.^[^ ^156^, ^157^ ^]^ Recent studies compellingly demonstrate that reactive astrocytes, exhibiting functional, morphological, and molecular modifications, play biphasic roles—either beneficial or detrimental—depending on the biological context. These effects are mediated through cell‐autonomous or non‐cell‐autonomous mechanisms after ischemic stroke.^[^ ^158^, ^159^ ^]^ These dynamic changes depend on astrocyte interaction with microglia, neurons, and oligodendrocytes.^[^ ^159^, ^160^ ^]^ Furthermore, reactive astrocytes release pro‐inflammatory cytokines, such as IL‐1β, IL‐6, and TNF‐α, actively participating in the inflammatory process after an ischemic stroke.^[^ ^161^, ^162^ ^]^ Transient inhibition of mitochondrial complex I, achieved through Ginsenoside Rb1, can reduce mitochondrial ROS production, thereby preventing astrocyte activation. The findings by Ni et al. suggested that the reactive astrocytes may be a promising target for pharmacological interventions for ischemic stroke.^[^ ^163^ ^]^ Additionally, studies indicate that reactive astrocytes post‐stroke can synthesize and release anti‐inflammatory cytokines and neurotrophic factors contributing to improved functional outcomes, infarct volume, neuronal function, synapses, and plasticity.^[^ ^164^, ^165^, ^166^ ^]^ In summary, the intricate interplay among microglia, astrocytes, and oligodendrocytes in response to ischemic stroke underscores a dynamic landscape in the restoration of neural tissue functioning. Targeting these reactive astrocytes emerges as a promising therapeutic avenue for interventions in ischemic stroke. Furthermore, the multifaceted functions of astrocytes, encompassing proinflammatory responses and the synthesis of neurotrophic factors, may play a pivotal role in influencing outcomes related to heart disease post‐stroke.

### 4.4 Gut Microbiome Dysbiosis

After an ischemic stroke in the elderly, over 50% of patients encounter gastrointestinal complications such as hemorrhage, dysphagia, constipation, and bowel incontinence. These complications are associated with poor patient outcomes, including delayed recovery times, deteriorated neurologic function, and increased mortality rates.^[^ ^167^, ^168^ ^]^ The intestinal epithelial barrier plays a crucial role in absorbing electrolytes, nutrients, and water while preventing pathogenic toxins and pathogens from the lumen into circulation, suggesting the interconnectedness between gut permeability and intestinal to systemic inflammation and metabolism.^[^ ^106^, ^169^, ^170^ ^]^ Gut permeability increases as early as 3 h post‐stroke, substantiated by quantifying serum levels of FITC‐labeled dextran gavage and reduced ZO‐1 in the ileum, leading to gut microbiome changes and dysfunction.^[^ ^167^ ^]^ Greater deficits in intestinal permeability of the brain–gut–microbiota axis result in more significant disruption of the central nervous system (CNS) consequences.^[^ ^106^, ^171^ ^]^ Accumulating evidence demonstrates that the intestinal microbiota plays a critical role in the pathophysiology and outcomes of ischemic stroke by altering the composition of the intestinal microbiota. Furthermore, many experimental and clinical studies have reported that the risk factors for stroke, such as hypertension, diabetes, and obesity, are associated with gut microbiota.^[^ ^171^ ^]^ The brain and gut are connected, forming a complex microbiota‐gut‐brain axis via a network of neurons, exhibiting strong bidirectional interactions. The gut microbiota contributes to the inflammatory response, thrombosis risk, and platelet hyperactivation through its waste production, trimethylamine N‐oxide (TMAO), associated with CVD and stroke.^[^ ^171^, ^172^, ^173^ ^]^ Some clinical trials also suggest that TMAO can be considered a marker and predictor for CVD.^[^ ^171^, ^174^ ^]^ Its levels participate in gut microbiota‐dependent changes and have been associated with myocardial infarction, atherothrombotic heart disease, endothelial cell activation, and ventricular remodeling.^[^ ^173^, ^175^, ^176^ ^]^ Moreover, plasma TMAO levels independently predict the risk of incident thrombotic events such as stroke and myocardial infarction.^[^ ^176^ ^]^

**Figure 2:**
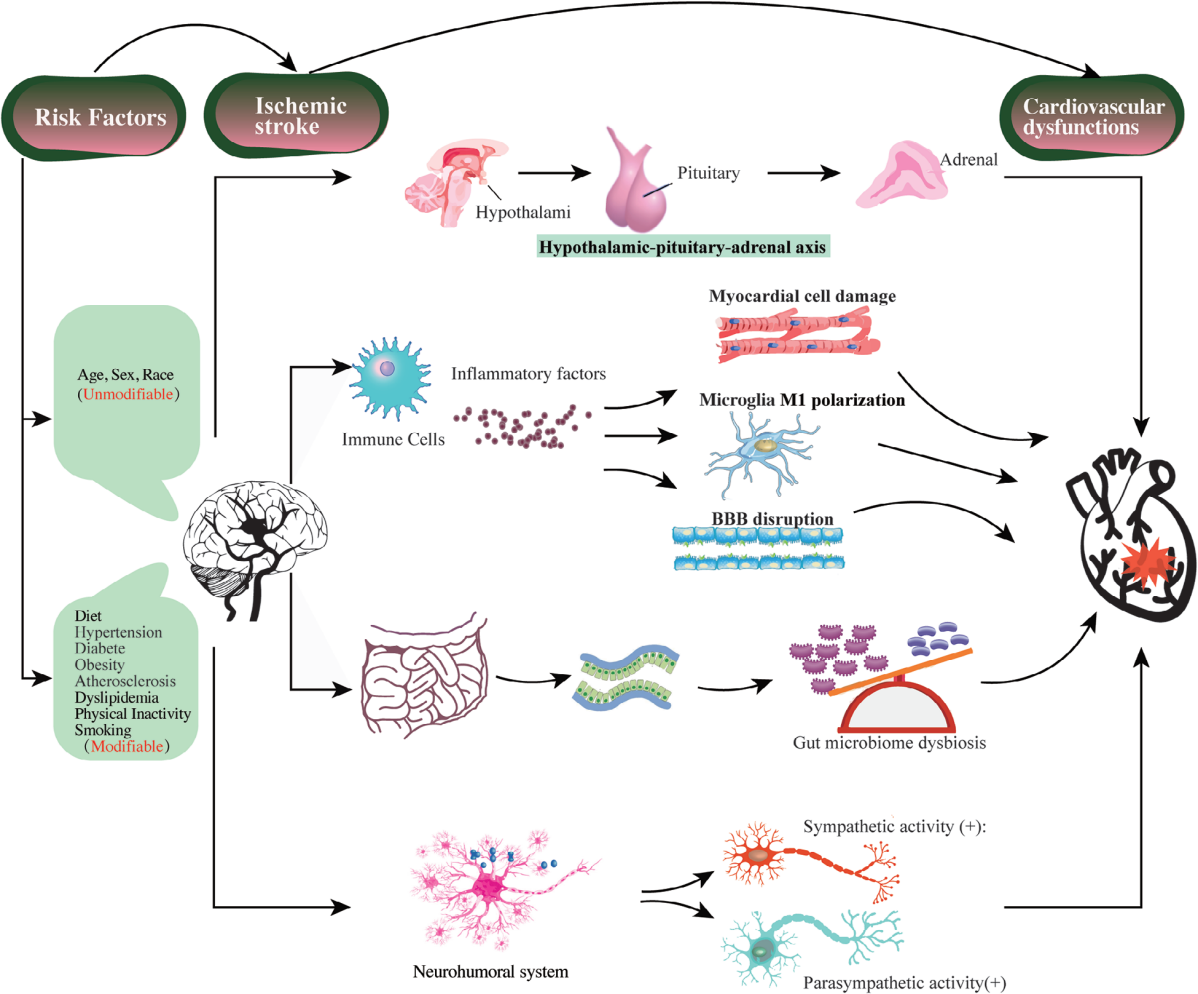
Mechanisms and related risk factors in stroke‐heart axis. The main mechanisms involved in the stroke‐heart crosstalk include the hypothalamic‐pituitary‐adrenal axis (HPA), inflammation, atherosclerosis, microbiota‐immune axis, and neurohumoral system. Some related risk factors are associated with stroke‐heart crosstalk. Abbreviations: BBB, blood‐brain barrier; DAMP, danger‐associated molecular patterns; TLPs, toll‐like receptors; TMAO, trimethylamine N‐oxide; CVD, cardiovascular diseases; BDNF, brain‐derived neurotrophic factor; PKB, protein kinase B; cAMP, cyclic adenosine monophosphate.

While there is accumulating preclinical and clinical research evidence about the mechanisms of stroke‐heart crosstalk, further investigation is still needed to uncover its exact underlying mechanisms. Identifying these mechanisms could provide promising therapeutic and preventive roles, promoting its use as a clinical therapy (**Figure** **2**).

## 5 Autoregulation Following AIS

**Figure 3:**
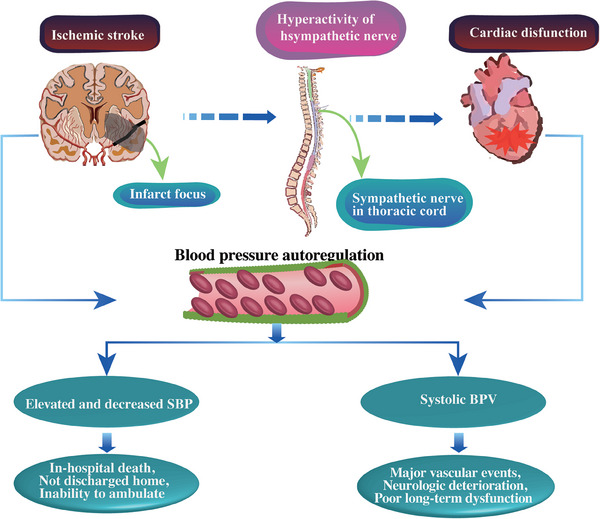
Blood pressure autoregulation in the brain–heart axis after AIS. Following AIS, the brain tissue in the basal ganglia region is injured, and the stroke brain displays the influence on the heart through the hyperactive sympathetic nerve in the thoracic cord. Blood pressure is commonly regulated by both the brain and heart after stroke. The elevated and decreased SBP and systolic BPV bring negative brain and heart outcomes. Abbreviations: SBP, systolic blood pressure; BPV, blood pressure variability.

Cerebral autoregulation plays a pivotal role in maintaining optimal cerebral perfusion pressure and blood flow following AIS, driven by physiological response or sympathetic hyperactivity.^[^ ^177^ ^]^ Addressing elevated SBP, a notable global health risk, becomes imperative for preventing adverse AIS outcomes.^[^ ^87^, ^177^ ^]^ A delicate equilibrium between the necessity for perfusion and the risk of hemorrhage poses a substantial challenge in AIS patients, distinct from autoregulation concerns. Numerous studies underscore the positive impact of BP control in AIS. Their findings indicate that both lower and higher SBP levels correlate with an elevated risk of in‐hospital death, failure to be discharged home, and an inability to ambulate independently upon discharge.^[^ ^178^ ^]^ Additionally, heightened systolic BPV, posing an increased stroke risk, strongly correlates with major vascular events, neurologic deterioration, and unfavorable long‐term functional outcomes post‐AIS.^[^ ^81^, ^179^, ^180^, ^181^ ^]^ A meta‐analysis revealed that while antihypertensive drugs significantly prevented recurrent stroke and cardiovascular events in individuals with a history of stroke or transient ischemic attack (TIA), they did not mitigate the risk of myocardial infarction or all‐cause mortality.^[^ ^182^, ^183^, ^184^ ^]^ Similarly, a meta‐analysis involving 12703 patients reported that lowering BP did not prevent death within 3 days of AIS onset.^[^ ^185^ ^]^ Research by Liu et al. advocated individualizing BP management based on the patient's condition.^[^ ^186^ ^]^ The American Heart Association (AHA) guidelines recommend initiating antihypertensive treatment in AIS if SBP exceeds 220 mmHg or diastolic blood pressure (DBP) surpasses 110 mmHg. Within the first 24 h post‐stroke onset, a 15% reduction in BP may be considered reasonable.^[^ ^187^ ^]^ The individualization of BP control remains crucial, aligning with the patient's unique clinical circumstances. Enhancing autoregulation post‐AIS between the brain and heart is beneficial in regulating adverse outcomes for both organs (**Figure** **3**).

## 6 Cardiovascular Monitoring and Treatment Options after AIS

Arrhythmic dysfunction is a common occurrence following AIS, recognized by inpatient neurologists, and consistently contributes to increased stroke morbidity and mortality.^[^ ^17^, ^75^, ^188^ ^]^ Cardiac ECG abnormalities, encompassing prolonged QTc, ST depression, AF, and T‐wave inversion, manifest in over 90% of AIS patients.^[^ ^20^, ^21^, ^37^, ^189^ ^]^ Arrhythmias, when coupled with acute hemodynamic dysfunction, compromise cerebral perfusion pressure, correlating with elevated post‐stroke mortality.^[^ ^1^, ^126^ ^]^

In a retrospective cross‐sectional study from Wira et al., AIS patients admitted from an urban emergency department were assessed within 24 h of symptom onset. Results indicated that troponin elevation, systolic dysfunction, AF, and ischemic changes on ECG may be linked to increased in‐hospital mortality rates, highlighting the importance of monitoring cardiac complications in AIS presentation.^[^ ^190^ ^]^ Furthermore, patients with a National Institutes of Health Stroke Scale/Score (NIHSS), a method of risk stratification for detecting the cardiovascular risk of AIS patients, exceeding 10 demonstrated more ischemic changes in ECG, elevated troponin levels, and a higher incidence of in‐hospital mortality compared with those with a score below 10.^[^ ^190^ ^]^ Early monitoring and analysis of ECG, along with assessing cardiac biomarkers and identifying patients at risk of acute cardiac events, are recommended. Therefore, we advocate for continuous ECG monitoring, maintaining electrolyte balance, and serial troponin sampling in patients with AIS.

Given the shared risk factors for cerebrovascular diseases and CVD, both the Pooled Cohort Equations and Framingham Risk Score offer valuable tools for assessing risks in patients concerning AIS and cardiovascular events, particularly when modifying related risk factors for treatment.^[^ ^110^, ^191^ ^]^

Additionally, acute myocardial infarction, a potentially life‐threatening complication, significantly impacts survival, cost of care, and hospital length of stay in patients admitted with AIS.^[^ ^59^, ^192^ ^]^ Serum levels of cardiac Troponin I (cTnI) or T (cTnT) serve as indicators of cardiac damage following ischemic stroke, and other brain injuries. cTnI is acknowledged as a more specific and sensitive biomarker for detecting cardiac damage and left ventricular (LV) dysfunction compared to creatine kinase‐MB (CK‐MB). Unlike CK‐MB, which is not entirely cardiac‐specific and may elevate in response to skeletal muscle injury, kidney failure, intramuscular injection, strenuous exercise, and exposure to various toxins and drugs, cTnI provides a more precise indication of cardiac involvement. Elevations in CK‐MB in patients with large hemispheric strokes are likely of non‐cardiac origin.^[^ ^193^ ^]^ Specific infarctions in brain regions, such as the right insula in AIS patients, are associated with serum cTnT elevation, indicating myocardial injury.^[^ ^194^ ^]^ Clinical studies have reported that elevated serum cTnT in AIS patients correlates with AIS severity in neurological deficits, damage to the insular lobe, worse prognosis, and an increased risk of death.^[^ ^195^, ^196^, ^197^ ^]^ A recent study revealed that patients who experienced AIS in the right hemisphere's dorsal anterior insular cortical region exhibited elevated relative changes in high‐sensitivity cTnT levels. These changes may contribute to autonomic disbalance, sympathetic activation, and myocardial injury.^[^ ^198^ ^]^

Serum brain natriuretic peptide (BNP) is highly linked to cardioembolic stroke, functional outcomes of AIS patients, and increased post‐stroke mortality.^[^ ^199^, ^200^, ^201^, ^202^ ^]^ The examination of cardiac enzymes proves significant in identifying patients at high risk of cardiovascular events following AIS. C‐reactive protein (CRP), indicative of inflammation, is a valuable marker for identifying increased CVD risk. A high‐sensitivity CRP level represents heightened cardiovascular risk and predicts CVD‐associated mortality for patients.^[^ ^203^, ^204^ ^]^

While consensus guidelines are available to guide clinical treatment decisions, the evidence supporting many recommended interventions following AIS still requires improvement. In severe cases, ICU management for AIS patients consistently emphasizes optimizing systemic physiological homeostasis, neurological and hemodynamic monitoring, and managing intracranial complications after reperfusion therapies.^[^ ^205^ ^]^ Continuous cardiac monitoring is crucial for detecting severe cardiac arrhythmias, especially AF, strokes related to cardiac embolism, and cardiovascular complications.^[^ ^206^, ^207^, ^208^, ^209^ ^]^

Oral anticoagulation (OAC) effectively prevents stroke occurrence and serves as the cornerstone for managing patients with AF.^[^ ^210^ ^]^ Recently, catheter ablation of AF, offering chronic safety and excellent durability, has been considered a more practical alternative to maintaining sinus rhythm, surpassing antiarrhythmic drug treatment.^[^ ^211^, ^212^, ^213^ ^]^ The LAA is recognized as a significant source of cardiac thrombus formation leading to stroke in AF patients. It has become an essential ablation target, particularly for AF patients on long‐term direct OAC at a high risk of bleeding and stroke.^[^ ^210^, ^214^, ^215^, ^216^, ^217^ ^]^ It is generally agreed that β‐blockers form the basis of medical therapy to reduce infarct size, mitigate sympathetic hyperactivity, increase LVEF, and prevent cardiac remodeling during the initial 24 hours following myocardial infarction onset.^[^ ^218^, ^219^ ^]^

In a large nonrandomized comparison involving 5212 patients, β‐blockers are reported to possess neuroprotective properties, reducing mortality and decreasing infectious complications after stroke. This suggests that randomized trials may be essential to investigate the potential of β‐blockers in acute stroke.^[^ ^220^ ^]^ Metoprolol, β1‐blocker, improved long‐term cardiac dysfunction in the preclinical setting by restraining higher sympathetic activity and alleviating extracellular cardiac remodeling in MCAO mice.^[^ ^99^ ^]^ A clinical study by Newton et al. indicated that nonselective β‐blockade may exert favorable inhibitory effects on cardiac sympathetic activity by antagonizing β‐adrenergic receptors in patients with heart failure.^[^ ^221^, ^222^ ^]^ However, a clinical study by Koton et al. suggested that pre‐stroke use of β‐blockers in hypertensive patients was not associated with functional outcomes, stroke severity, or death assessed using the NIHSS or the European Stroke Scale (ESS) and modified Rankin scale (mRS) score.^[^ ^223^, ^224^ ^]^ A meta‐analysis reported that the suggested beneficial effects of β‐blockers were not substantiated based on the results obtained during the acute phase of stroke in more than 100 000 patients.^[^ ^225^ ^]^ High‐quality further research should investigate the mechanisms of β‐blockers in the context of CVD and AIS.

Lenz et al. observed that the intravenous administration of ST‐115 displayed a protective effect in mitigating both ischemic and reperfusion injury, alleviating bradykinin degradation to increase bradykinin's concentration in capillaries in a mouse model of myocardial infarction and a rat model of ischemic stroke. It is suggested that the successful combination of thrombectomy and intravenous ST‐115 administration could simultaneously reduce ischemic and reperfusion injury after a stroke, indicating potential clinical utility.^[^ ^226^ ^]^

In various models of myocardial infarction, fibroblast growth factor (FGF) signaling demonstrates a cardioprotective function with its receptors (FGFRs) expressed in multiple heart cell types. Inactive FGFRs in Tie2‐Cre, Fgfr1(f/f), and Fgfr2(f/f) DCKO mice revealed significantly worsened cardiac function compared to controls at day 7 after reperfusion. Pathophysiological analysis of the peri‐infarct area revealed significantly increased endothelial cell apoptosis, reduced vessel density, and aggravated tissue hypoxia. These findings suggest a potential therapeutic benefit through activating endothelial FGFRs after ischemic heart injury.^[^ ^227^ ^]^ FGF21 treatment improved microglia/macrophage‐mediated neuroinflammation, promoting functional recovery through the NF‐κB and PPAR‐γ signaling pathways. This suggests a potential anti‐inflammatory strategy for mice with AIS.^[^ ^228^ ^]^ In parallel, intranasal administration of nonmitogenic FGF1 facilitated functional recovery by modulating microglia/macrophage‐mediated neuroinflammation through Nrf2 and NF‐κB signaling pathways in AIS mice. These findings were corroborated in in vitro experiments, indicating that nonmitogenic FGF1 holds promise as an agent against ischemic stroke.^[^ ^229^ ^]^

The NLRP3 (NLR pyrin domain‐containing 3) inflammasome serves as a crucial component in the inflammatory response following stroke.^[^ ^230^ ^]^ Lin et al. discovered that the cardiac dysfunction induced by AIS is more severe in a type 2 diabetes mice model. Notably, they found that the NLRP3 inflammasome is primarily activated in M1 macrophages, and its inhibitor CY‐09 can restore cardiac function. This suggests that the activation of M1 macrophage‐NLRP3 inflammasome is an underlying mediator of the brain–heart interaction following diabetic AIS.^[^ ^67^, ^231^ ^]^ Subsequent to splenectomy in mice with stroke, there was a reduction in neurological function impairment, macrophage infiltration into the heart, expression of inflammatory factors in the heart, cardiac hypertrophy, and fibrosis. Additionally, cardiac function significantly improved compared to stroke mice without splenectomy. These findings suggest that the immune response induced by cerebral ischemic stroke may contribute to post‐stroke cardiac dysfunction.^[^ ^232^, ^233^ ^]^

In the context of myocardial inflammation, apoptosis, and cardiac dysfunction induced by stroke, chronic exercise has been found to confer protection via improving APJ expression and inhibiting p‐STAT3 in the MCAO stroke mice compared with acute exercise.^[^ ^234^ ^]^ Numerous clinical studies have reported that the concurrent release of TnT and an increased incidence of systemic inflammatory response syndrome coincided with cardiac dysfunction in patients with AIS.^[^ ^235^, ^236^ ^]^ Additionally, in the preclinical research, Vornholz et al. reported that reperfusion after ischemic stroke leads to specific cardiocirculatory alterations characterized by acute heart failure with bradycardia and changes in cardiac tissue. These alterations are accompanied by systemic and local inflammatory responses within the myocardial tissue of AIS mice.^[^ ^237^ ^]^

Additionally, cardiac dysfunction can be induced by stroke even in the absence of primary cardiac diseases. Chen et al. conducted a study where differentially expressed genes were identified by RNA sequencing of mRNA, mainly distributed in the membrane or extracellular region of cardiomyocytes. These genes acted as potential mediators of stroke‐induced cardiomyopathy and cardiac dysregulation‐associated cardiac atrophy of experimental MCAO mice hearts.^[^ ^238^, ^239^ ^]^

**Figure 4:**
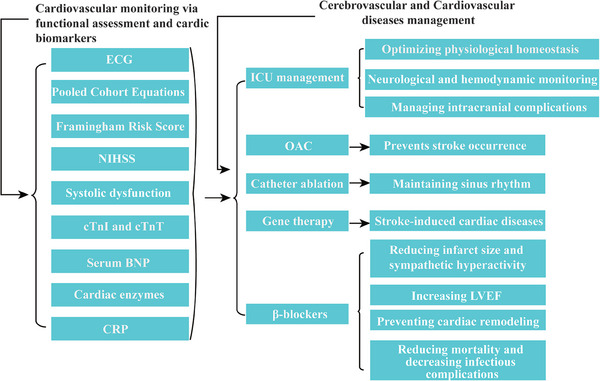
Cardiovascular monitoring and treatment options following AIS. Following AIS, kinds of monitoring methods can be utilized for detecting cardiovascular disease. Moreover, some recommended methods are provided for managing cerebrovascular and cardiovascular diseases. Abbreviations: ECG, electrocardiogram; cTnI or T, cardiac troponin I or T; NIHSS, National Institutes of Health Stroke Scale; BNP, brain natriuretic peptide; CRP, C‐reactive protein; ICU, Intensive care unit; OAC, Oral anticoagulation; LVEF, left ventricular ejection fraction.

The substantial body of evidence strongly indicates that incorporating cardiovascular monitoring plays a pivotal role in implementing positive therapeutic interventions for managing heart diseases induced by AIS. Vigilant monitoring of cardiovascular events is crucial for effectively mitigating the adverse outcomes after an AIS (**Figure** **4**).

## 7 Conclusion

The interaction between the heart and brain is multifactorial and complex, necessitating further elucidation. Despite significant progress in comprehending brain–heart crosstalk following AIS, the roles of inflammation, gut dysbiosis, and sympathetic control remain unclear. The exploration of these aspects in stroke–heart interactions is a promising theme that continues to captivate researchers’ interest. Delving deeper into the accurate molecular mechanisms representing pharmacological targets within these pathways is crucial. This exploration holds the potential to modulate the brain–heart axis and interrupt their related pathogenetic transmission, paving the way for promising individualized therapeutical strategies.

Moreover, the underlying causative mechanisms associated with stroke‐induced heart dysfunction still require further investigation, and novel targets will be explored for future therapeutic strategies. Continued studies are necessary to explore these intriguing mechanisms and translate these theories into clinical applications. Persistent investigation into the causality and directionality of brain–heart interactions holds promise in the evolving field of neurocardiology in the coming years.

## Conflict of Interest

The authors declare no conflict of interest.

## Author Contributions

Conceptualization: I.A., X.Z., G.Y., X.F.; Writing—Original Draft Preparation, X.F., G.Y.; Writing—review and editing, J.C., M.L., D.Z., I.A., I.E.‐B.; Visualization, X.Z., G.Y., X.F., G.C.; Funding Acquisition, X.Z., I.A., G.Y., X.F.; All authors have read and agreed to the published version of the manuscript.
